# 5hmC modification regulates R-loop accumulation in response to stress

**DOI:** 10.3389/fpsyt.2023.1198502

**Published:** 2023-06-09

**Authors:** Xingyun Xu, Junjie Wang, Wenjuan Wang, Yutong Zhang, Bo Wan, Zhigang Miao, Xingshun Xu

**Affiliations:** ^1^Department of Neurology, The First Affiliated Hospital of Soochow University, Soochow University, Suzhou, China; ^2^Institute of Neuroscience, Soochow University, Suzhou, China; ^3^Jiangsu Key Laboratory of Neuropsychiatric Diseases, Soochow University, Suzhou, Jiangsu, China

**Keywords:** R-loop, 5-hydroxymethylcytosine, ten-eleven translocation, stress, NONO

## Abstract

R-loop, an RNA-DNA hybrid structure, arises as a transcriptional by-product and has been implicated in DNA damage and genomic instability when excessive R-loop is accumulated. Although previous study demonstrated that R-loop is associated with ten-eleven translocation (Tet) proteins, which oxidize 5-methylcytosine to 5-hydroxymethylcytosine (5hmC), the sixth base of DNA. However, the relationship between R-loop and DNA 5hmC modification remains unclear. In this study, we found that chronic restraint stress increased R-loop accumulation and decreased 5hmC modification in the prefrontal cortex (PFC) of the stressed mice. The increase of DNA 5hmC modification by vitamin C was accompanied with the decrease of R-loop levels; on the contrary, the decrease of DNA 5hmC modification by a small compound SC-1 increased the R-loop levels, indicating that 5hmC modification inversely regulates R-loop accumulation. Further, we showed that Tet deficiency-induced reduction of DNA 5hmC promoted R-loop accumulation. In addition, Tet proteins immunoprecipitated with Non-POU domain-containing octamer-binding (NONO) proteins. The deficiency of Tet proteins or NONO increased R-loop levels, but silencing Tet proteins and NONO did not further increase the increase accumulation, suggesting that NONO and Tet proteins formed a complex to inhibit R-loop formation. It was worth noting that NONO protein levels decreased in the PFC of stressed mice with R-loop accumulation. The administration of antidepressant fluoxetine to stressed mice increased NONO protein levels, and effectively decreased R-loop accumulation and DNA damage. In conclusion, we showed that DNA 5hmC modification negatively regulates R-loop accumulation by the NONO-Tet complex under stress. Our findings provide potential therapeutic targets for depression.

## Introduction

R-loop, a three-stranded structure, emerges as a transcriptional byproduct. The nascent RNA strand recombines with the DNA template strand, forming a DNA:RNA hybrid, and this hybrid strand forms an R-loop structure with the non-template DNA single strand (1–4). R-loop accumulation induces stalled replication forks, forming a barrier to transcription and DNA replication, which has an important association with genomic instability (5). R-loops predominantly localize within GC-rich genomic regions and are implicated in various neurological disorders such as but not limited to characterized by multinucleated repeat expansions in genes (6–11). TAR DNA-binding protein 43 (TDP43) deficiency-induced R-loop accumulation has been associated with TDP-43 proteinopathy *via* DNA replication stress (11). In cellular models of C9orf72 repeat amplification, multiple repeats containing either r(GGCCCC) or r(GGGGCC) induce augmented R-loop accumulation (9). Additionally, abnormal RNA:DNA hybrid accumulation is observed in Aicardi–Goutières syndrome patients (6). The regulatory mechanisms underlying R-loop accumulation remain elusive.

Recent research suggests a connection between ten-eleven translocation (Tet) enzymes and R-loop accumulation (12). Tet enzymes oxidize 5-methylcytosine (5mC) to 5-hydroxymethylcytosine (5hmC) in the presence of cofactors such as Fe(II), oxygen, and α-ketoglutarate-dependent dioxygenase (13–15). 5hmC plays a pivotal role in neurodevelopment and neurodegenerative disorders by facilitating active DNA demethylation and exhibiting high enrichment within the nervous system (16). Abnormal 5hmC modifications are detected in numerous neurological disorders, such as Alzheimer’s disease, where hippocampal neurons display reduced 5hmC levels that inversely correlate with amyloid plaque load (17). Our previous work in mouse models demonstrated that stress-induced dynamic 5hmC alterations in the hippocampus influence gene expression (18). 5hmC was found to recruit endonucleases to degrade stalled replication forks (19) and accumulates at sites of DNA damage and repair (20), suggesting a role in maintaining genomic integrity. The relevance of 5hmC modification to R-loop accumulation through Tet enzymes is yet to be determined.

An increasing body of evidence demonstrates that the epigenetic factor is one of the mechanisms of depression (21, 22). According to previous studies, Tet proteins are related the phenotypes of depression in mice under stress (18, 23). It is unclear whether Tet enzymes crosstalk with R-loop in the depressive conditions and that antidepressants have effects on R-loop accumulation. In this study, we demonstrated that stress significantly increased R-loop accumulation. Tet deficiency-mediated 5hmC reduction exhibited a negative correlation with R-loop accumulation. Furthermore, we found that Tet interacted with NONO to inhibit R-loop accumulation. Interestingly, antidepressant fluoxetine reduced R-loop accumulation in depressive mice.

## Materials and methods

### Animals

Male ICR mice (15–25 g) were purchased from Jihui Company (Shanghai, China) and housed under specific-pathogen-free conditions at the Soochow University Experimental Animal Center. The mice were exposed to a 12 h light-dark cycle and provided *ad libitum* access to water and food. All behavioral tests were conducted between 9:00–12:00 am during the light phase, with mice acclimatized to the testing environment for 2 h beforehand. All experimental mice were male at the age of 8–16 weeks. Animal care and experimental procedures adhered to National Regulations for the Administration of Affairs Concerning Experimental Animals and were approved by the Institutional Animal Care and Use Committee of Soochow University.

### Chronic restraint stress

Male mice were randomized into control and chronic restraint stress (CRS) groups. CRS group mice were individually confined in modified, ventilated 50 mL centrifuge tubes for 2 h (9:00–11:00 am) daily for 3 weeks, preventing forward or backward movement. Stressed mice were returned to their cages after 2 h restraint periods. Control group mice experienced unrestricted movement in their cages, albeit without access to water and food.

### Tail suspension test

Mouse tails were affixed to a rod approximately 35 cm above a desktop, suspending the mice for 6 min. Struggle behaviors were recorded. The immobility time, defined as the duration of trunk, limb, and head immobility, was measured within the 6 min period.

### Forced swim test

Mice were placed in a 2 L beaker containing 1.6 L of water (23°C–25°C) and filmed from above for 6 min. The immobility time, defined as the duration of floating and motionlessness, was measured during the entire 6 min interval.

### Drugs

Fluoxetine, acquired from Selleck Chemicals (United States), was dissolved in 0.9% saline and intraperitoneally administered to mice at a dose of 20 mg/ (kg·d) for 3 weeks, with saline serving as the control. SC-1 acquired from Cayman Chemical (United States), was dissolved in DMSO. Vitamin C acquired from Solarbio (China), was dissolved in phosphate buffer saline (PBS).

### Western blot analysis

Tissues and cells were harvested and lysed using cell lysis buffer (FUDE Biological Technology, China) containing protease inhibitors on ice for a minimum of 30 min. Following 15 min centrifugation at 4°C, supernatants were collected, combined with loading buffer, and heated for 10 min at 100°C. Proteins were resolved using 8% or 12% sodium dodecyl sulfate–polyacrylamide gel electrophoresis and transferred onto polyvinylidene fluoride membranes. Membranes were subsequently blocked with 5% non-fat milk for 1 h at room temperature and incubated with primary antibodies overnight at 4°C. Primary antibodies targeting Tet1 (1:1000, Gene Tex, United States), Tet2 (1:1000, Abcam, United Kingdom), Tet3 (1:1000, Gene Tex, United States), NONO (1:10000, Santa Cruz Biotechnology, United States), and β-actin (1:10000, HUAAN, China) were employed. On day two, membranes were incubated with horseradish peroxidase-conjugated secondary antibodies for 2 h at room temperature. Protein signals were detected *via* an enhanced chemiluminescence system, and band intensities were analyzed using Image Lab software (Bio-Rad, United States).

### Immunofluorescence staining

Mice were sequentially perfused with pre-cooled saline and 4% paraformaldehyde, followed by immersion of mouse brains in 4% paraformaldehyde overnight at 4°C. Mouse brains were gradiently dehydrated in sucrose solutions and sliced into 20 μm sections using a cryostat. Sections were permeabilized with 0.2% Triton X-100 in phosphate-buffered saline (PBST) and blocked with 0.2% Triton X-100 and 2% bovine serum albumin (BSA) in PBS for 1 h at room temperature. Primary antibodies, including NeuN (1:500, Abcam, United Kingdom), 5hmC (1:500, Active Motif, United States), S9.6 (1:5000, Kerafast, United Kingdom), and Tet3 (1:100, Abcam, United Kingdom), were incubated with sections overnight at 4°C. Sections were subsequently incubated with secondary antibodies (1:300, Jackson ImmunoResearch Laboratories, United States) for 2 h at room temperature, and images were acquired using a confocal microscope (Zeiss, Germany).

### Cell culture and plasmid transfection

Hippocampal neuronal (HT22) cells were cultured in Dulbecco’s Modified Eagle Medium (DMEM) supplemented with 10% fetal bovine serum, 100 UI/mL penicillin sodium, and 100 μg/mL streptomycin sulfate. Cells at 50%–60% confluency were transfected with plasmids using polyethylenimine (PEI, Polysciences, United States) as a transfection reagent in Optional Minimal Essential Medium (Opti-MEM) for 20–30 min, followed by addition to the culture medium. Cells were harvested for further analysis after 48 h.

### Short hairpin RNA and small interfering RNA knockdown

Tet1-short hairpin RNA (shRNA): 5′-CCGGTTTCAACTCCGACGTAAATATCTCGAGATATTTACGTCGGAGTTGAAATTTTTG-3′, Tet3-1-shRNA: 5′-CCGGGAACCTTCTCTTGCGCTATTTCTCGAGAAATAGCGCAAGAGAAGGTTCTTTTTG-3′, Tet3-2-shRNA: 5′-CCGGGCTCCAACGAGAAGCTATTTGCTCGAGCAAATAGCTTCTCGTTGGAGCTTTTTG-3′ and control empty shRNA (pLKO.1) were used lentiviral transfections, performed in the presence of polybrene (8 μg/mL). Small interfering RNA (siRNA) knockdown was conducted according to previously published protocols (24). Tet2 siRNA sequence was 5′-GGAUGUAAGUUUGCCAGAATT-3′. NONO siRNA sequence was 5′-AGAGGGCUGUAGUCAUUGUG-3′ and control siRNA sequence was 5′-UUCUCCGAACGUGUCACGUTT-3′. siRNA sequences were synthesized by Sangon Biotech (Shanghai, China). Cells were transfected with Lipofectamine RNAiMAX (Invitrogen, United States) and siRNA. The mixture was incubated in Opi-MEM for 20–30 min and added into the culture medium. Cells were harvested for further analysis after 48 h.

### Dot blot for R-loop detection

Genomic DNA was extracted from samples lysed at 55°C using a DNA lysis solution (1 M Tris pH 8.0, 0.5 M EDTA pH 8.5, 5 M NaCl, 10% SDS) containing 0.2 mg/mL proteinase K, following a phenol-chloroform method. Genomic DNA was precipitated with isopropanol, washed with 75% ethanol, and resuspended in DNase/RNase-free water. For the negative control, 1 μg of genomic DNA was digested with RNase H (Thermo Scientific, United States) at 37°C for 2 h. Another 1 μg of genomic DNA was digested with RNase T1 (Thermo Scientific, United States) and RNase III (New England Biolabs, United States) for 2 h at 37°C. Genomic DNA treated with 2 N NaOH was spotted on nitrocellulose membranes, baked for 30 min at 80°C, and blocked with 5% non-fat milk in PBS. The membranes were incubated with R-loop antibody S9.6 overnight at 4°C, followed by incubation with secondary antibodies the next day. R-loop signals were detected using an enhanced chemiluminescence system, and images were captured. Spot intensities were analyzed with Image Lab software (Bio-Rad, United States).

### Immunoprecipitation assay

Samples were fully lysed in cell lysis buffer containing protease inhibitors (FUDE Biological Technology, China) and centrifuged at high speed at 4°C. Supernatants were incubated with protein A/G magnetic beads (Bimake, United States) or anti-Flag magnetic beads (Bimake, United States) for 6–12 h at 4°C. Protein A/G magnetic beads and anti-Flag magnetic beads were pre-blocked with 0.5% bovine serum albumin/PBS for 2 h, and protein A/G magnetic beads were pre-coupled with specific antibodies using a rotary instrument for 6–12 h at 4°C. After washing with cell lysis buffer, immunoprecipitants and loading buffer were boiled for 10 min at 100°C. 10% of the supernatant was served as input. Immunoprecipitants were further analyzed by western blot.

### R-loop immunoprecipitation assay

The R-loop immunoprecipitation method, previously described (25), involves capturing genomic R-loops with S9.6 antibody, followed by detection of R-loop-interacting proteins *via* western blot assay. Tissue samples were lysed using FA lysis solution and genomic DNA was fragmented into 500–1,000 bp fragments by sonication. Following centrifugation, supernatants were collected. Protein A/G magnetic beads, pre-blocked with 0.5% BSA/PBS for 2 h, were incubated with S9.6/IgG antibodies for 6–12 h at 4°C, with IgG antibody serving as a control. Supernatants were incubated with protein A/G magnetic beads for 6–12 h at 4°C, and RNase T1 and RNase III were added during the immunoprecipitation process. After washing, immunoprecipitants and loading buffer were boiled for 10 min at 100°C. 2% of the supernatant was served as input. Immunoprecipitants (IP) and input were analyzed by western blot. To detect R-loops, immunoprecipitants were eluted in 1% SDS and 0.1 M NaHCO3 for 30 min at room temperature for dot blot analysis.

### Statistics

Data were expressed as the mean ± standard deviation (SD) and analyzed using GraphPad Prism software 8 (GraphPad, San Diego, CA). Two-tailed unpaired Student’s *t*-test was used to compare the differences between the two groups. Differences among groups were analyzed by two-way ANOVA with *post hoc* test. A *p*-value of less than 0.05 was considered statistically significant.

## Results

### CRS induced R-loop accumulation in the PFC

**Figure 1:**
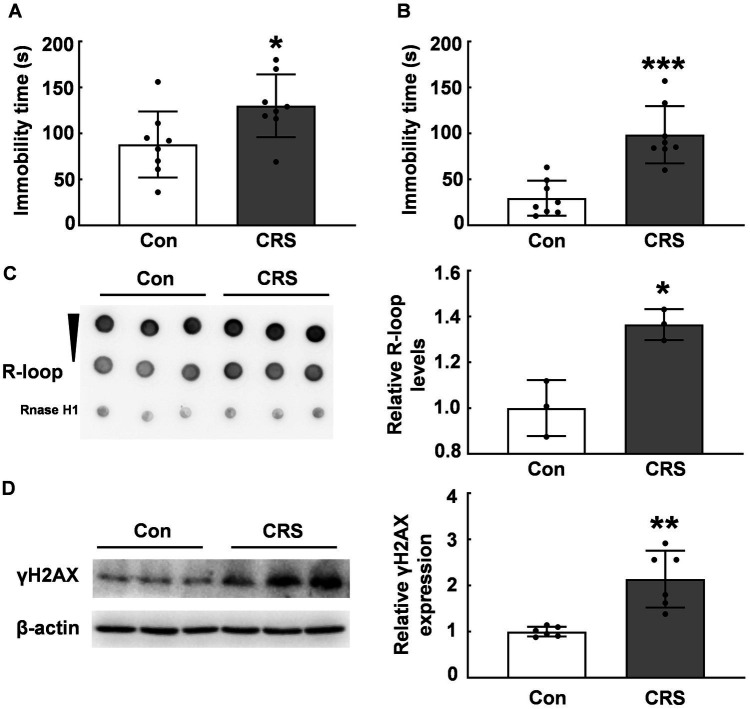
R-loop accumulation and DNA damage in CRS mice. **(A)** In forced swim test (FST), immobility time in male CRS mice was compared with that in control mice (*N* = 8; *p* = 0.0306). **(B)** In tail suspension test (TST), immobility time was examined in male CRS mice and control mice (*N* = 8; *p* = 0.0001). **(C)** R-loop levels in the prefrontal cortex (PFC) of CRS and control mice were measured by dot blot. RNase H treatment used as a negative control (*N* = 3; *p* = 0.0106). **(D)** γH2AX protein levels in the PFC of CRS mice and control mice were determined by western blot (*N* = 6; *p* = 0.0012). *p < 0.05; **p < 0.01; ***p < 0.001.

Previous research has established that abnormal R-loop metabolism contributes to DNA damage and genomic instability, which is related to neurological diseases, including Huntington’s, Fragile X, myotonic dystrophy type 1, and Aicardi–Goutières syndrome (6, 26). However, the impact of environmental stress on R-loop formation remains unclear. We employed the CRS model to examine R-loop levels in mouse brain tissues. Under CRS, stressed mice exhibited significantly increased immobility times in forced swim test and tail suspension test compared with those of control mice (*p* < 0.05, Figures 1A,B). We assessed R-loop levels in the PFC of CRS mice and control mice and found a significant increase of R-loop levels in CRS mice (*p* < 0.05, Figure 1C), suggesting R-loop accumulation in response to stress. γH2AX protein levels, a marker of DNA damage, were markedly increased in stressed mice compared with those of control mice (*p* < 0.01, Figure 1D). This was consistent with the notion that R-loop causes genomic instability accompanied with DNA damage.

### DNA 5hmC modification inversely regulated R-loop accumulation

**Figure 2:**
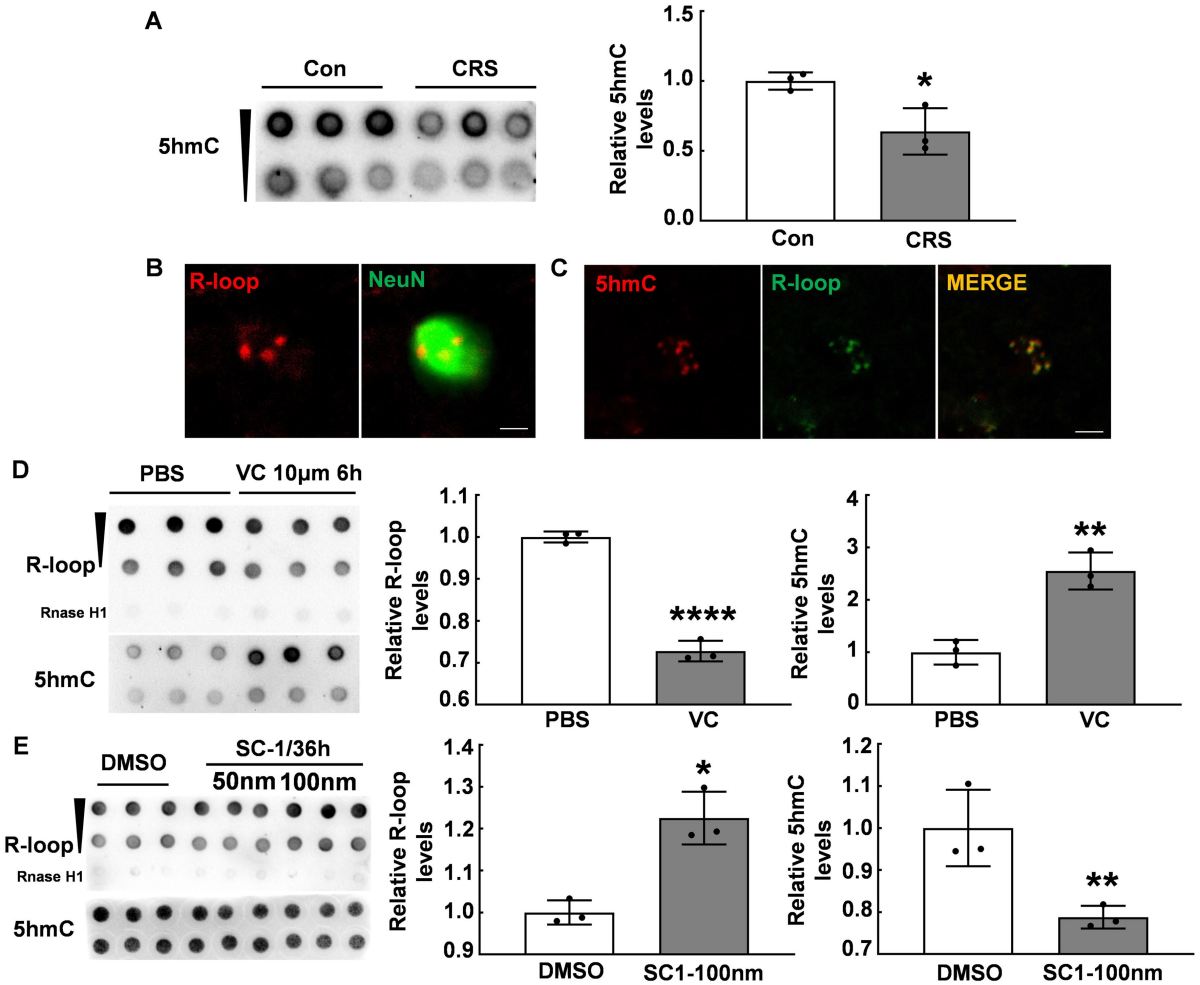
DNA 5hmC modification inversely regulated R-loop accumulation. **(A)** 5hmC levels in the PFC of CRS mice and control mice detected by dot blot (*N* = 3; *p* = 0.0244). **(B)** Immunofluorescent staining of R-loop and NeuN (a neuronal marker) in male mouse PFC was shown. Scale bar: 5 μm. **(C)** Immunofluorescent staining of 5hmC and R-loop in male mouse PFC was shown. Scale bar: 5 μm. **(D)** R-loop (*p* ≤ 0.0001) and 5hmC (*p* = 0.0031) levels in HT22 cells treated with VC (10 μm, 6 h) and PBS were assessed by dot blot (*N* = 3). **(E)** R-loop (*p* = 0.0049) and 5hmC (*p* = 0.0181) levels in HT22 cells treated with SC-1 and DMSO for 36 h were evaluated by dot blot (*N* = 3). *p < 0.05; **p < 0.01; ***p < 0.0001.

Given previous reports indicating an association between Tet enzymes and R-loop accumulation (12), we examined the global DNA 5hmC modifications in the PFC of CRS mice and control mice using the dot blot assay. Our data showed a significant reduction in the global DNA 5hmC modifications in the PFC of stressed mice (*p* < 0.05, Figure 2A), consistent with previous findings (18). In addition, immunofluorescent staining showed R-loop expression in NeuN^+^ positive cells (Figure 2B), aligning with abundant 5hmC expression in neurons (27). To investigate the relationship between DNA 5hmC and R-loops, we observed 5hmC and R-loop localization by immunofluorescent staining, and revealed their co-localization (Figure 2C). We subsequently used vitamin C (VC), a co-factor of Tet enzymes, to elevate the global DNA 5hmC modification levels. Our results showed the global 5hmC levels substantially increased in the VC group compared with that in the control group (*p* < 0.01, Figure 2D), however, there was a concurrent significant decrease in R-loop levels (*p* < 0.0001, Figure 2D). Further, the cells were treated with a small compound SC-1, an inhibitor of Tet enzymes, and markedly decreased the global DNA 5hmC (*p* < 0.01, Figure 2E), but significantly increased R-loop levels (*p* < 0.05, Figure 2E). Therefore, these findings suggest that 5hmC modification inversely regulates R-loop accumulation.

### Tet enzymes regulated R-loop accumulation

**Figure 3:**
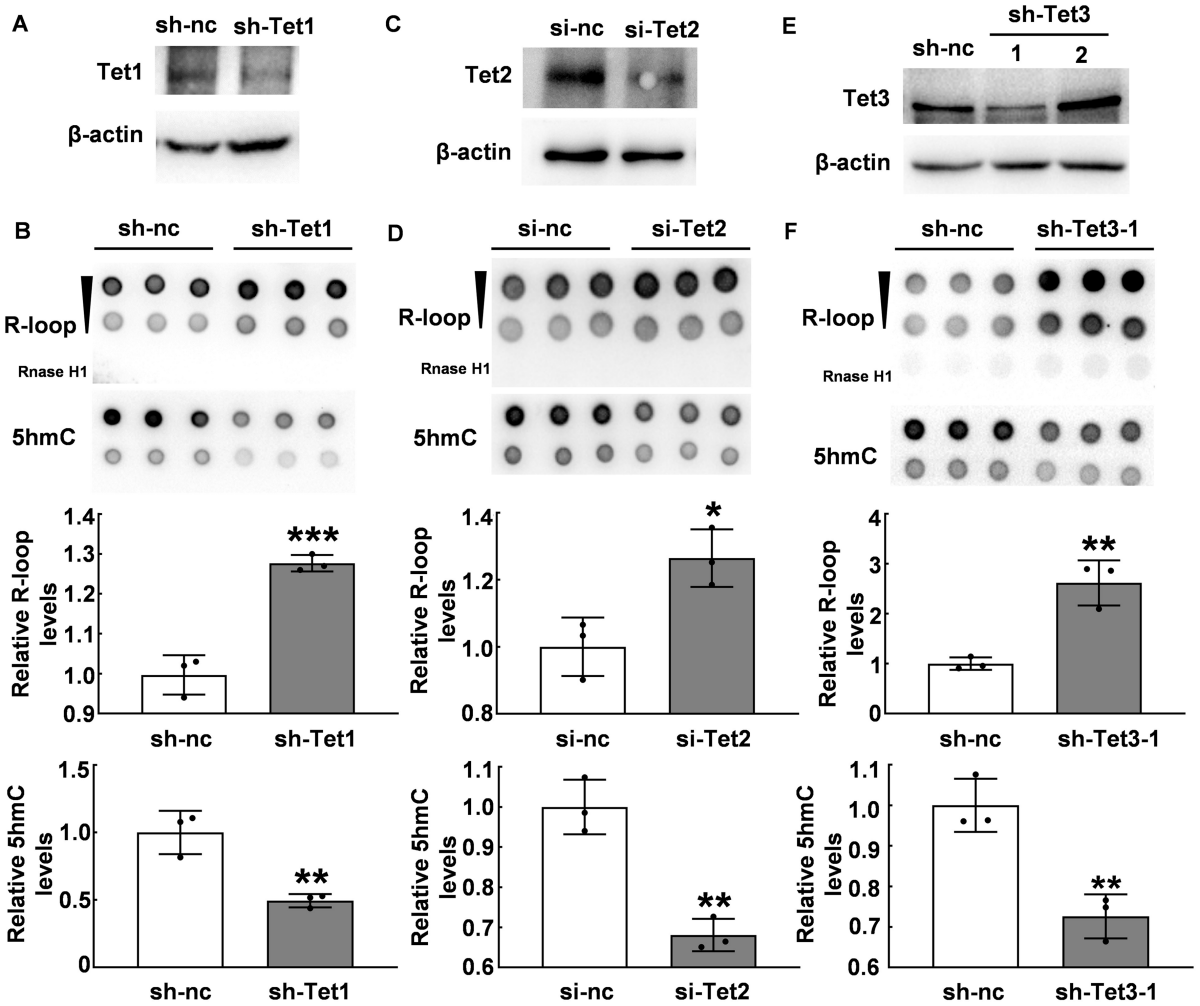
Tet proteins modulated R-loop accumulation. **(A)** Tet1 was silenced with Tet1 shRNA in HT22 cells for 48 h and Tet1 protein was examined by western blot. **(B)** R-loop (*p* = 0.0008) and 5hmC (*p* = 0.0064) levels in HT22 cells with Tet1 shRNA for 48 h was evaluated by dot blot (*N* = 3). **(C)** Tet2 was silenced in HT22 cells by using Tet2 siRNA for 48 h. Tet2 protein levels was examined by western blot. **(D)** R-loop (*p* = 0.02) and 5hmC (*p* = 0.0022) levels in HT22 cells with Tet2 siRNA for 48 h were assessed by dot blot (*N* = 3). **(E)** Tet3 was silenced in HT22 cells with Tet3 shRNA for 48 h. Tet3 protein levels was examined by western blot. **(F)** R-loop (*p* = 0.0039) and 5hmC (*p* = 0.0051) levels were detected by dot blot in HT22 cells with Tet3 shRNA for 48 h (*N* = 3). *p < 0.05; **p < 0.01; ***p < 0.001.

Due to the inverse correlation between 5hmC modification and R-loop levels, we further investigated whether Tet enzymes regulate R-loop accumulation. We suppressed Tet1 expression by using shTet1 lentivirus infection. Our western blot results revealed a significant decrease in both Tet1 protein (Figure 3A) and 5hmC levels (*p* ≤ 0.01, Figure 3B). Interestingly, R-loop levels significantly increased after Tet1 knockdown (*p* < 0.001, Figure 3B). We also used Tet2 siRNA to knock down Tet2 (Figure 3C) and observed a significant decrease in 5hmC levels and a significant increase in R-loop levels compared with those of control group (*p* < 0.05, Figure 3D). Similarly, we tested Tet3 protein in cells infected with shTet3 lentivirus and found that Tet3 (Figure 3E) and 5hmC levels (*p* ≤ 0.01, Figure 3F) significantly reduced in shTet3 lentivirus-treated cells. Knockdown of Tet3 also induced a significant increase in R-loop level (*p* < 0.01, Figure 3F). These findings suggest that Tet enzymes regulate R-loop accumulation.

### NONO and Tet proteins formed a complex in brain tissues

**Figure 4:**
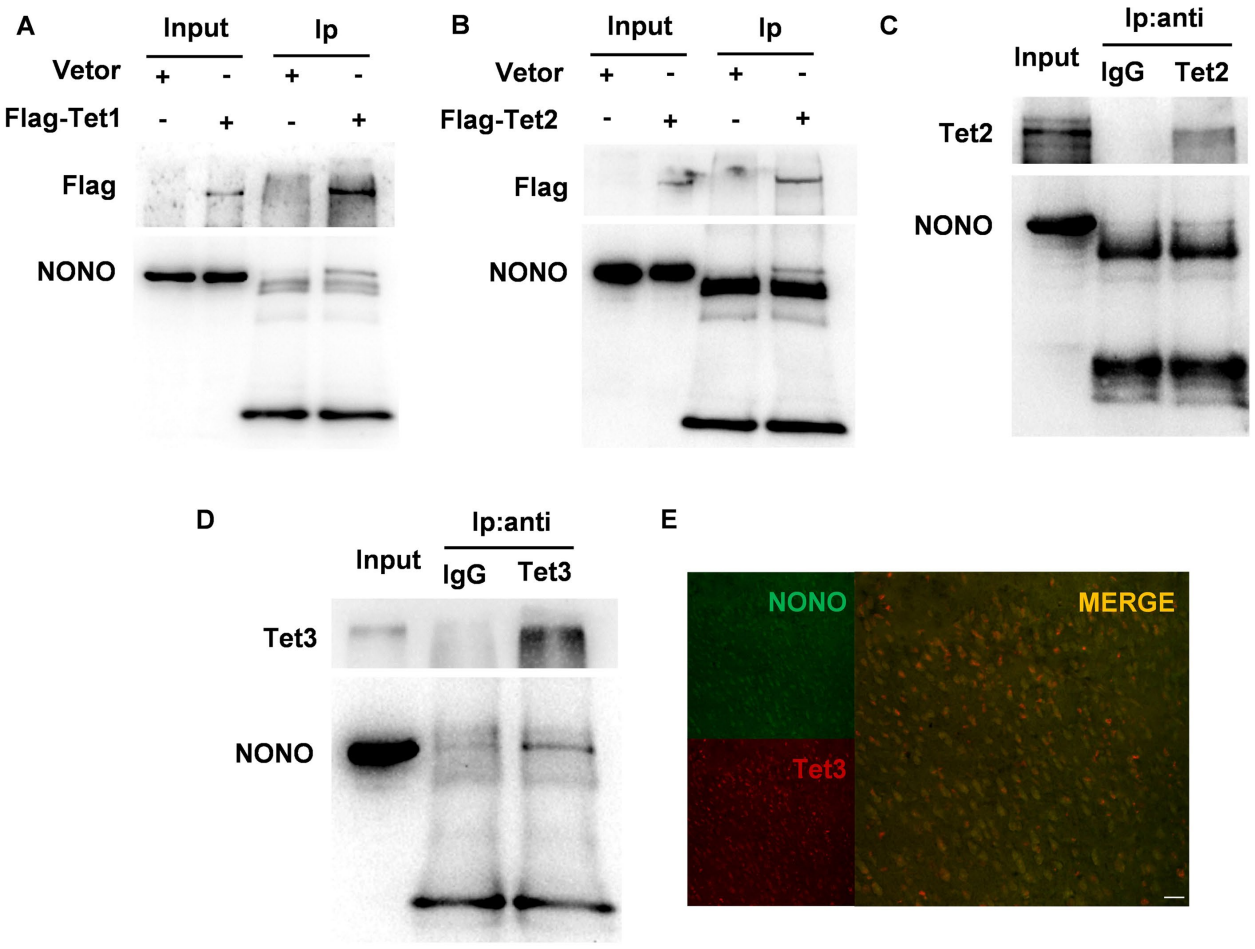
NONO, Tet, and R-loop formed an interacting complex. **(A)** HEK293 cells were transfected with vector or Flag-Tet1 for 48 h. Immunoprecipitation analysis of cell lysates was performed by using anti-Flag magnetic beads. **(B)** HEK293 cells were transfected with vector or Flag-Tet2 for 48 h. Immunoprecipitation analysis of cell lysates was performed with anti-Flag magnetic beads. **(C)** Mouse cortex lysates were subjected to immunoprecipitation assay by using anti-Tet2 antibody or control IgG. **(D)** Mouse cortex lysates were subjected to immunoprecipitation assay with anti-Tet3 antibody or control IgG. **(E)** Immunofluorescent staining of Tet3 and NONO in the male mouse cortex was shown. Scale bar: 20 μm.

Previous research has demonstrated that NONO interacts with Tet1 and modulates Tet1 activity (28). We performed co-immunoprecipitation using Flag-tag magnetic beads to precipitate Flag-Tet1 and NONO. Western blot results showed the presence of NONO and Tet1 in the precipitates, as depicted in Figure 4A, suggesting the interaction of NONO with Tet1. We further detected the interaction of NONO and Tet2 by the similar method, showing that there was a more pronounced interaction between NONO and Flag-Tet2 (Figure 4B). Also, endogenous NONO and Tet2 interactions were assessed, with NONO protein detection in mouse cortical tissue Tet2-immunoprecipitated proteins (Figure 4C). Similarly, we pulled the proteins down with anti-Tet3 antibody in the cortex of mice and found the presence of NONO in Tet3-immunoprecipitated proteins (Figure 4D). Moreover, NONO was co-distributed in the nucleus with Tet3 in the cortex (Figure 4E). These findings suggest NONO forms complexes with Tet proteins.

### The NONO-Tet complex negatively regulated the accumulation of R-loop

**Figure 5:**
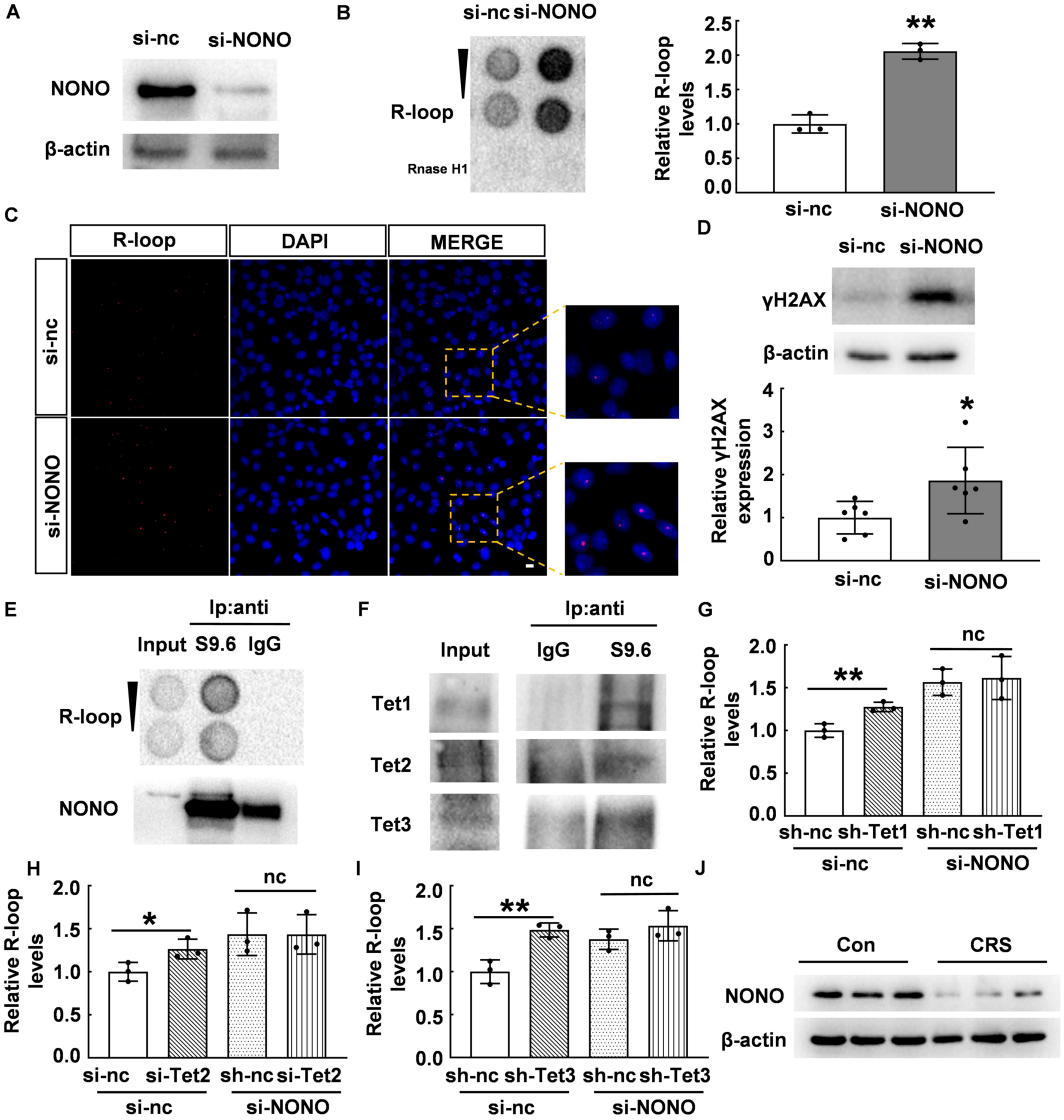
NONO-Tet interaction influenced R-loop accumulation. **(A)** HT22 cells were transfected with control or NONO siRNAs for 48 h. NONO protein levels were assessed by western blot. **(B)** HT22 cells were transfected with control or NONO siRNAs for 48 h. R-loop levels were measured by dot blot (*N* = 3; *p* = 0.0005). **(C)** HT22 cells were transfected with control or NONO siRNAs for 48 h. Immunofluorescent staining of R-loop and DAPI were displayed. Scale bar: 20 μm. **(D)** HT22 cells were transfected with control or NONO siRNAs for 48 h. γH2AX protein levels were examined by western blot (*N* = 6; *p* = 0.0334). **(E,F)** R-loop immunoprecipitation assay was performed in mouse cortex by using anti-S9.6 antibody or control IgG. **(E)** R-loop and NONO levels were detected. **(F)** Tet protein levels was determined by western blot. **(G)** HT22 cells were transfected with control, Tet1 shRNA, and NONO siRNA as indicated for 48 h. R-loop dot density in dot blot was analyzed (*N* = 3; *p* = 0.0077). **(H)** HT22 cells were transfected with control, Tet2, and NONO siRNAs as indicated for 48 h. R-loop levels was examined by dot blot. R-loop dot density was analyzed (*N* = 3; *p* = 0.0415). **(I)** HT22 cells were transfected with control, Tet3 shRNA, and NONO siRNA as indicated for 48 h. R-loop dot density in dot blot was analyzed (*N* = 3; *p* = 0.0061). **(J)** The protein levels of NONO in the PFC of CRS and control mice were detected by western blot (*N* = 3). *p < 0.05; **p < 0.01; ns > 0.05.

NONO acts as a multifunctional nuclear protein involved in DNA unwinding (29, 30), transcriptional activation (31, 32), transcriptional termination (33), and other processes. We hypothesized that NONO plays a crucial role in the regulation of R-loop accumulation. To verify this, we knocked down NONO by siRNA and confirmed NONO protein expression by western blot as shown in Figure 5A. We observed a significant increase in R-loop levels in NONO-deficient cells (*p* < 0.01, Figure 5B). Consistently, immunofluorescence staining also supported the increase of R-loop in NONO-deficient cells (Figure 5C), while NONO knockdown also markedly upregulated γH2AX protein levels (*p* < 0.05, Figure 5D), indicating NONO silencing-induced DNA damage.

To elucidate the role of the NONO-Tet complex in R-loop accumulation, we performed immunoprecipitation using anti-S9.6 antibody (R-loop-specific) (25) to examine potential interacting proteins. Dot blot analysis showed R-loop detection in the immunoprecipitated complex with anti-S9.6 antibody, but not with anti-IgG antibody (Figure 5E). NONO was found to be co-immunoprecipitated with R-loop in mouse cortex samples (Figure 5E). Crucially, Tet proteins were detected in the immunoprecipitated complex (Figure 5F). Considering the NONO-Tet interaction, these results indicate that NONO, Tet, and R-loop form an interacting complex, suggesting NONO-Tet complex is associated with R-loop formation.

Given the observed interaction and complex formation between NONO and Tet enzymes, we further explored their potential relationship with R-loop accumulation. Tet1 knockdown in HT22 cells induced R-loop accumulation, similar to NONO knockdown (*p* < 0.01, Figure 5G). However, Tet1 silencing did not further exacerbate R-loop accumulation resulting from NONO knockdown (*p* > 0.05, Figure 5G). Likewise, Tet2 or Tet3 knockdown also led to R-loop accumulation (*p* < 0.01, Figures 5H,I), but their silencing did not further increase the accumulation induced by NONO knockdown (*p* > 0.05, Figures 5H,I). This suggests that NONO and Tet proteins inhibit R-loop formation through NONO-Tet complex, and that the absence of either of the complex components affects the complex function, resulting in abnormal R-loop accumulation. In addition, we examined the protein levels of NONO in the PFC of CRS mice and found a significant reduction of NONO protein (Figures 5J), suggesting that NONO plays an essential role in R-loop accumulation in CRS mice.

### Fluoxetine alleviated R-loop accumulation in CRS mice

**Figure 6:**
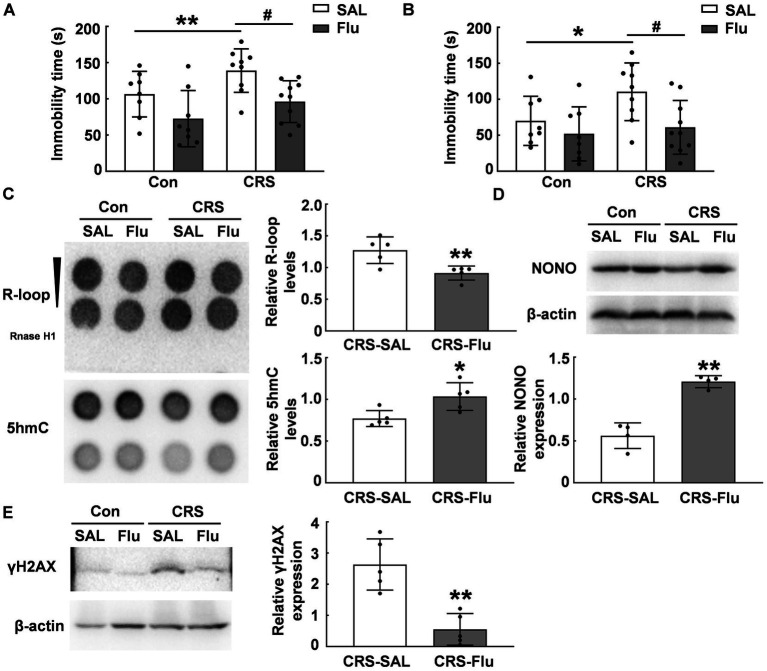
Fluoxetine treatment alleviated R-loop accumulation in CRS mice. **(A.B)** ICR mice were subjected to chronic restraint stress daily for 3  weeks, followed by 3  weeks of saline or fluoxetine treatment. Behavioral tests **(A)** FST (*N* = 8–10; *p*, * = 0.0011, *p*, # = 0.0403) and **(B)** TST were conducted (*N* = 8–10; *p*, * = 0.0151, *p, #* = 0.0345). **(C)** 5hmC (*p* = 0.02) and R-loop (*p* = 0.006) levels were assessed in the PFC of CRS mice treated with saline or fluoxetine (*N* = 5). **(D)** NONO protein levels were determined in the PFC of CRS mice treated with saline or fluoxetine by using western blot (*p* = 0.0018; *N* = 4). **(E)** γH2AX protein levels were determined in the PFC of CRS mice treated with saline or fluoxetine by using western blot (*p* = 0.0051; *N* = 5). *p < 0.05; **p < 0.01; #*p* < 0.05.

Fluoxetine is a widely-used antidepressant in the clinical for patients. We treated CRS mice with fluoxetine and found a reduction in immobility time during FST (*p* < 0.05, Figure 6A) and TST (*p* < 0.05, Figure 6B) compared with those CRS mice treated with saline. Fluoxetine treatment increased 5hmC modification levels in the PFC of CRS mice (*p* < 0.05, Figure 6C) and attenuated R-loop accumulation induced by CRS (*p* < 0.01, Figure 6C). Further, fluoxetine treatment increased NONO protein levels in the PFC of CRS mice (*p* ≤ 0.01, Figure 6D). Additionally, fluoxetine administration reduced γH2AX protein levels in the PFC caused by CRS (*p* < 0.01, Figure 6E). These findings suggest NONO may be a potential target for antidepressants.

## Discussion

In this study, we demonstrated a causal link between 5hmC modification and R-loop accumulation. We found that DNA 5hmC modifications decreased under CRS simultaneously accompanying the increase of R-loop accumulation. Furthermore, we identified a negative association between R-loop accumulation and 5hmC modifications. Interestingly, the NONO/Tet complex negatively regulates R-loop accumulation.

### DNA 5hmC modification regulates R-loop accumulation

5hmC, an epigenetic modification marker of DNA damage (20), may exhibit cell-specific and time-dependent effects on genomic stability regulation. Some studies have shown that Tet enzymes catalyze DNA 5hmC modifications responding to DNA damage and promote genome integrity (12, 20, 34), while others have reported that 5hmC modifications induce abnormal DNA replication and γH2AX accumulation (35). Our results demonstrate that stress induced the decrease in the level of 5hmC modification (Figure 2A), which supports the previous report that stress reduces the distribution of 5hmC in the PFC of mice (18), and the increase of γH2AX levels and R-loop accumulation. Our findings revealed 5hmC modification negatively regulates R-loop accumulation and DNA damage, which connects the two different epigenetic modifications. Tet acts as an α-ketoglutarate and Fe^2+^-dependent dioxygenase to catalyze the conversion of 5mC to 5hmC (36), therefore, Tet enzymes may affect R-loop formation. Although recent studies have implicated the association of Tet proteins and R-loop formation (12, 37), the effects of Tet proteins on R-loop formation are still unclear. One study reported that Tet2 and Tet3 deficiency increases G-quadruplex and R-loop formation and promotes B-cell lymphoma development in mice (12). Another study found that Tet1/2/3 triple knockdown reduces global R-loop levels in mouse fibroblasts (37). These discrepancies may be due to the different cell lines and different conditions. Therefore, the relationship between Tet proteins and R-loop formation needs more investigations.

### The NONO-Tet complex is necessary for R-loop formation

NONO, an RNA and DNA binding protein, is involved in various biological processes, such as transcriptional regulation (38), DNA repair (39–41), RNA splicing (42), RNA silencing (43), and nuclear mRNA retention (44). Prior research has shown that NONO deficiency results in telomere replication defects in U2OS cells, implicating the critical role of NONO in maintaining genome stability (45). Recent study has revealed that NONO recruits Tet1 through direct interactions to contribute to neuronal differentiation in mouse embryonic stem cells (28). In this study, our findings confirmed that NONO interacted not only with Tet1 but also with Tet2 and Tet3. Importantly, our results demonstrate that the NONO-Tet complex negatively regulates R-loop accumulation. Moreover, the absence of Tet or NONO leads to abnormal R-loop accumulation, but both Tet and NONO silencing did not further exacerbate R-loop accumulation. These suggest that NONO-Tet complex is essential for the R-loop formation. NONO is associated with human neurodevelopment, and hemizygous loss-of-function variants cause neurodevelopmental disorders, accompanied by intellectual disability, cognitive, and emotional deficits (46). Our study demonstrate that the NONO-Tet interaction is involved in stabilizing R-loop. In this study, depression-like mice exhibited abnormal R-loop accumulation in the PFC of mice and NONO protein levels were reduced in depressive mice. Importantly, fluoxetine treatment effectively increased the protein levels of NONO. This provides evidence that the NONO-Tet complex is important to improve the emotional deficits of depression by antidepressants.

In summary, we demonstrate that DNA 5hmC modification negatively regulates R-loop accumulation by the NONO-Tet complex. Our study provides evidence to connect two important epigenetic modifications DNA 5hmC modification and R-loop together. NONO-Tet-mediated 5hmC modification is negatively associated with R-loop accumulation and DNA damage in both cells and depressive mice. This study may provide potential therapeutic targets for depression.

## Data availability statement

The original contributions presented in the study are included in the article/supplementary material, further inquiries can be directed to the corresponding authors.

## Ethics statement

The animal study was reviewed and approved by the Institutional Animal Care and Use Committee of Soochow University.

## Author contributions

XsX and ZM designed experiments and revised the manuscript. XyX, JW, and WW performed the experiments and analyzed the data. BW and ZM discussed the data. XyX drafted the manuscript. All authors contributed to the article and approved the submitted version.

## Conflict of interest

The authors declare that the research was conducted in the absence of any commercial or financial relationships that could be construed as a potential conflict of interest.

## Publisher’s note

All claims expressed in this article are solely those of the authors and do not necessarily represent those of their affiliated organizations, or those of the publisher, the editors and the reviewers. Any product that may be evaluated in this article, or claim that may be made by its manufacturer, is not guaranteed or endorsed by the publisher.
